# Benefits of Home-Based Solutions for Diagnosis and Treatment of Acute Coronary Syndromes on Health Care Costs: A Systematic Review

**DOI:** 10.3390/s20175006

**Published:** 2020-09-03

**Authors:** Pau Redón, Atif Shahzad, Talha Iqbal, William Wijns

**Affiliations:** 1 CÚRAM Center for Research in Medical Devices, H91 W2TY Galway, Ireland; william.wijns@nuigalway.ie; 2 Smart Sensor Lab, School of Medicine, National University of Ireland, Galway (NUIG), H91 TK33 Galway, Ireland; atif.shahzad@nuigalway.ie (A.S.); t.iqbal1@nuigalway.ie (T.I.); 3 Saolta University Healthcare Group, University Hospital Galway, Newcastle Road, H91 YR71 Galway, Ireland

**Keywords:** cardiovascular disease, acute myocardial infarction, home-based solutions, troponin, cardiac biomarkers

## Abstract

Diagnosing and treating acute coronary syndromes consumes a significant fraction of the healthcare budget worldwide. The pressure on resources is expected to increase with the continuing rise of cardiovascular disease, other chronic diseases and extended life expectancy, while expenditure is constrained. The objective of this review is to assess if home-based solutions for measuring chemical cardiac biomarkers can mitigate or reduce the continued rise in the costs of ACS treatment. A systematic review was performed considering published literature in several relevant public databases (i.e., PUBMED, Cochrane, Embase and Scopus) focusing on current biomarker practices in high-risk patients, their cost-effectiveness and the clinical evidence and feasibility of implementation. Out of 26,000 references screened, 86 met the inclusion criteria after independent full-text review. Current clinical evidence highlights that home-based solutions implemented in primary and secondary prevention reduce health care costs by earlier diagnosis, improved patient outcomes and quality of life, as well as by avoidance of unnecessary use of resources. Economical evidence suggests their potential to reduce health care costs if the incremental cost-effectiveness ratio or the willingness-to-pay does not surpass £20,000/QALY or €50,000 limit per 20,000 patients, respectively. The cost-effectiveness of these solutions increases when applied to high-risk patients.

## 1. Introduction

Over the past few decades, cardiovascular diseases (CVD) are in a continuous rise and currently accounting for 17.64 million deaths worldwide in the general population [1]. Moreover, CVD is the leading cause of death in patients with chronic kidney disease (CKD) and other comorbidities. In adult diabetic patients the probability of dying from heart disease is 2–4 times higher compared to their healthy peers [1].

**Figure 1:**
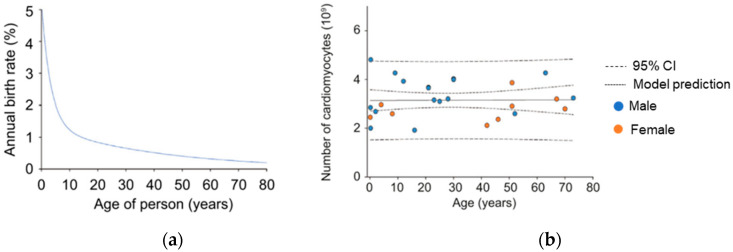
“Regeneration rate and number of cardiomyocytes depending on: (**a**) age and (**b**) gender” Olaf Bergmann et al. [3], licensed under the number 4772391132781.

One of the most common acute coronary syndromes (ACS) triggered by CVD are heart attacks, also known as acute myocardial infarction (AMI). These can be symptomatic or asymptomatic and are caused by a complete (ST-segment elevation MI, or STEMI) or partial blockage of coronary arteries (non-STEMI or unstable angina). The damage or injury is generated by the lack of blood and oxygen supply to the downstream myocardium and it can be largely irreversible in the absence of essential repair mechanisms, inherent to the myocardium. In healthy hearts, see Figure 1a, the regeneration rate of the heart cells decreases considerably with age [2,3] and equally affects males and females [3]. Focusing on cardiomyocytes, responsible for the contraction of the myocardium, their regeneration rate reduces from an annual rate of 5%, in the first years of life, to less than 0.5% in an elderly population. Consequently, as illustrated in Figure 1b, the number of cardiomyocytes remains approximately constant throughout the whole lifespan of males and females, without opportunity for repair in case of cell loss due to STEMI.

**Figure 2:**
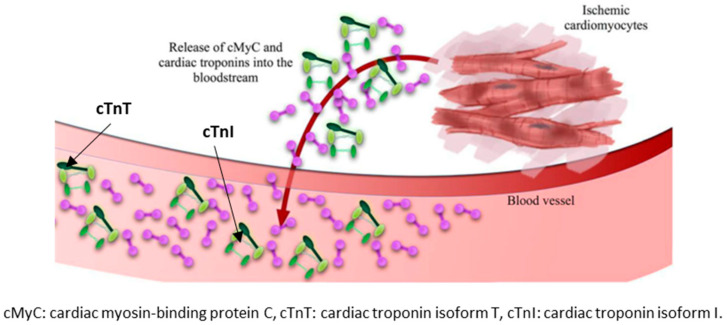
Depiction of cardiac troponin and cardiac myosin-binding protein and their release during myocardial injury by Twerenbold et al. [9], licensed under open access terms.

Independently from the type of AMI, the detection in the blood stream of abnormal levels of biomarkers associated with myocardial necrosis, in particular troponins, is now the standard evaluation and classification criterion according to the recently published Fourth Universal Definition of Myocardial Infarction Consensus Document [4]. This consensus document elaborated by several scientific associations (European Society of Cardiology (ESC), American Heart Association (AHA) and American College of Cardiology (ACC)) introduces several updates with respect to prior versions [5,6,7]. The most relevant observation in the consensus document is related to the differentiation between infarction and injury based on troponin levels. The troponin, a contractile protein, which is responsible for the heart’s contraction−relaxation movements, can be found as part of the cardiomyocyte structure in the isoforms I (cTnI) and T (cTnT). A troponin release into the circulatory system is a sign of ischemic cardiomyocytes or myocardial damage, see Figure 2. Currently, no other cardiac biomarker has shown higher diagnostic and prognostic capability than troponins [8,9,10].

Unfortunately, the incidence of AMI is expected to continue increasing with the rise of CVD and other chronic diseases (e.g., diabetes, hypertension and obesity) in conjunction with a rapidly aging population in the Western world. Such increase is expected to negatively impact the budget of national health care systems. Treating acute cardiac events, like AMI, consumes a significant fraction of the limited budget of the healthcare providers (UK €1.9 billion, France €1.3 billion, Germany €3.3 billion, Italy €3.1 billion, Spain €1.0 billion and US $12.1 billion), attributable to both the frequency with which it occurs and the cost associated with each acute hospitalization [10,11]. Even though a detailed analysis of these costs reveals that most of them are not modifiable by the care team or the health system, some potential reductions can be achieved by replacing routine-based practices with need-based practices [12]. This is especially relevant for high-risk patients (e.g., diabetic patients, elderly, heart failure and CKD) who demand quick and safe diagnostic procedures to reduce their risk of suffering recurrent events or dying. Consequently, interventions targeting these high-risk patients are more likely to yield effective results when it comes to reducing health care expenditure [13].

In this context, one potential solution worth considering is the use of home-based or telemonitoring systems, which are capable of measuring troponin levels in biological samples, easily implementable in current clinical practice and focusing on high-risk patients. The objective of this review is to explore these opportunities by assessing the available evidence in key enabling fields: sensing technologies currently available for home-based telemonitoring systems; relevance of personal data for diagnosis and prognosis of ACS including NSTEMI and STEMI; anticipated clinical benefits from implementation of telemonitoring in this context; and features that make an ambulatory troponin-based strategy potentially cost-effective.

## 2. Materials & Methods

**Figure 3:**
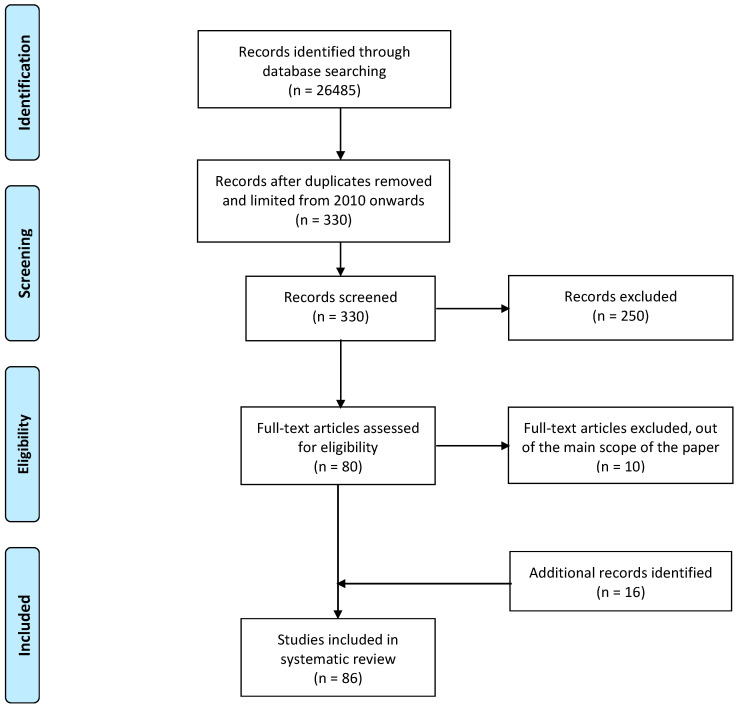
Schematic summary of the number of papers evaluated for this review according to the PRISMA methodology [14].

The Preferred Reporting Items for Systematic Reviews and Meta-Analyses (PRISMA) methodology [14] was used to synthesize the current evidence available in the published literature on home-based solutions for ACS management, see Figure 3. The major research databases, PubMed, Cochrane Library, Embase and Scopus, were scanned between the 1st of December 2019 and the 31st of January 2020.

The search terms were the result of combining 3 general keywords (cardiac troponin or cTn, Emergency Department (ED), diagnosis, prognosis, myocardial injury) with a maximum of 3 specific keywords (elderly, diabetes, CKD, heart failure, cost-effectiveness, economical, clinical, home-based or wearables or biosensors).

A total of 26,485 papers were initially retrieved after adding up the different combinations of the search terms and 330 were selected after removing duplicates, those not available in English and those published prior to 2010. Two-hundred and fifty papers were excluded during the title and abstract screening process and deemed irrelevant to the topic of this review. The excluded papers have focused on pathophysiological and biochemical principles as well as a different population compared to the high-risk population covered in this paper. The last step prior to final inclusion was to determine the eligibility of the 80 remaining references. A full-text analysis was performed on all of the selected 80 papers with special attention to identify the study’s limitations. In those cases, where this information was not available, the authors applied specific quality assessment tools, specially AGREE-II [15,16], see Table S1 in the Supplementary Materials section (Supplementary Material 1) for the list of items reviewed by this methodology. Based on the quality assessment, an additional 10 papers were excluded due to either important limitations with respect to selection bias (e.g., patient recruitment in clinical trials not performed randomly) or outcome bias (e.g., the results of not all measured variables were published) or because they were not published in first or second quartile journals. Only when a limited number of references were available for a certain topic, these were complemented by papers published in journals of the third or fourth quartile and by others identified from the reference list of already included papers. At the end, a total of 86 references were included in this systematic review, see Figure S1 included in the Supplementary Materials section (Supplementary Material 1) for further details.

The limitations of this review article can be potentially related to selection bias caused by three key aspects: (1) the selection of the search terms and how they are combined. Other combinations of these search terms may result in a slightly different search base; (2) the date and type of the databases scanned, including the list of journals indexed in these databases, which means some of the relevant manuscripts published in other journals or stored in other databases are expected to be omitted; and (3) only published data were considered, limiting the inclusion of more recent and updated studies pending publication.

## 3. Diagnosing AMI

### 3.1. Diagnosis and Prognosis of Cut-Off Values in Troponin-Based Assays

Sensitive (s-Tn) and high-sensitive (hs-Tn) troponin-based assays coexist commercially either integrated in bulky and expensive laboratory equipment or as a bedside point of care (PoC) solution [17]. The difference between them is not restricted to the limit of detection (LOD) of the assays but more importantly with the potential strategies these assays can drive. The hs-Tn assays can contribute to improve patient outcomes by reducing the event-to-diagnosis-time through their capacity to detect smaller changes in the concentrations of troponins between two consecutive readings. A key milestone regarding the recognition of this technology has been the presentation of the 0/1 h algorithm in the recently published ESC guidelines [8]. Despite this potential benefit their clinical implementation is still restricted probably due to some identified limitations: nonspecific elevations, false-positive results, biological variability and lack of assay standardization [18]. In high-risk patient cohorts (e.g., CKD, adult diabetic patients, heart failure (HF) and elderly patients) which demand rapid and more accurate diagnosis, these and other limitations can have major health care consequences.

#### 3.1.1. Chronic Kidney Disease

Focusing on the CKD cohorts of patients with CVD, important conclusions can be extracted from several recently published review papers [19,20]. Michos et al. [19] examined 124 studies to evaluate how troponin levels contributed to diagnosis, patient management and prognosis of ACS patients as well as risk stratification of patients without ACS symptoms. The results revealed that elevated cTnI or cTnT are potent predictors of mortality in CKD patients with and without suspicion of ACS and independent of the condition whether the patient is receiving dialysis or not. The authors suggested that measuring troponin levels may be reasonable for additional risk stratification. Stacy et al. [20] evaluated 23 trials where troponin levels were measured in CKD cohorts. The results revealed a need to identify optimal cut-off points of troponins for patients with CKD and ACS for proper risk stratification. Aligned with these conclusions is the study performed by Skadberg et al. [21]. The author measured, using hs-Tn assays, troponin levels before and after hemodialysis and found that mean values decreased during this process. Consequently, the author recommends the need to determine specific cut-off points, before and after hemodialysis, when using hs-Tn assays. Even though Twerenbold et al. [22] results were in agreement with the previous studies, they emphasized that optimal cut-off values should be assay-specific, isoform-specific and manufacturer-specific. In a multicenter study on 2813 patients, of which 16% had renal dysfunction, the conclusions were: (i) hs-Tn assay showed slightly lower diagnosis accuracy at presentation in CKD patients versus patients without renal dysfunction; (ii) no systematic superiority was found between hs-Tn and s-Tn assays; and (iii) hs-Tn loses specificity when comparing CKD with normal renal function patients.

#### 3.1.2. Diabetes

In diabetic patients, special emphasis goes to type 2 diabetes mellitus (T2DM) patients who are more common than type 1 diabetes mellitus (T1DM) in a 10:1 ratio. The ARIC [23] and the EXAMINE trial [24], with respectively 1500 and 3808 T2DM patients, pointed out that troponin testing using hs-Tn assays could be a good biomarker for personalized medicine. The ARIC study concluded that abnormal troponin levels can predict the CVD risk of T2DM patients 10 years in advance. The EXAMINE study found that elevated troponins can help to identify patients at extreme risk for cardiac events. The extreme risk for cardiac events is a new category identified in 2017 by the Association of Clinical Endocrinologists and American College of Endocrinology (AACE) on the Comprehensive T2D Management Algorithm. Both findings can contribute to improve the management of T2DM patients with more aggressive primary or secondary preventive interventions. Nonetheless, further studies are required to confirm this complementary valuable information. Surprisingly, meta-analyses have revealed that in most clinical settings, hs-Tn has a comparable diagnostic and prognostic performance to that of s-Tn [25].

#### 3.1.3. Elderly

The elderly population, with increasing life expectancy, is another high-risk cohort due to comorbidities and chronic diseases. Using hs-Tn in this cohort can be misleading because the pathophysiological mechanisms resulting in cardiomyocyte injury in the aging heart are still not well-understood and troponins can be elevated due to non-cardiovascular comorbidities (e.g., sepsis, myocarditis, drug toxicity, pulmonary embolism, hypoxia and global hypoperfusion) [26,27]. For example, in elderly NSTEMI patients, using the uniform assay-specific 99th percentile for STEMI diagnosis, troponins were shown to have limited diagnostic capability due to reduced specificity [28,29]. In this context, age-adjusted cut-off values [30,31] or even algorithm-specific [32] would be desirable. Nevertheless, to establish these cut-off values further research studies are required to determine the trade-off between diagnosis efficacy and prognosis capability.

#### 3.1.4. Heart Failure

The last high-risk cohort discussed in this paper is the HF patients. This life-threatening scenario can be reached due to recurrent or big injuries resulting from a prolonged delay, from event to intervention, producing massive losses of cardiomyocytes. Even though left ventricular ejection fraction contributes to the risk stratification of HF patients, the measuring of cardiac biomarkers (e.g., troponin (cTn) and brain natriuretic peptide (BNP) in biological fluids (e.g., blood, urine and saliva) are also useful in this population for diagnosis and management purposes [33,34] as well as to prognosticate for mortality and need for hospital readmission [34,35]. The ADHERE study [36] with 65,180 patients revealed that higher troponin levels were associated with higher in-hospital mortality. In the EFFECT study [37], with 2000 patients hospitalized for HF in Ontario (Canada), it was found that a troponin value greater than 0.5 μg/L during the first 48 h of hospitalization was a predictor of increased 1-year all-cause mortality. Other small scale studies [38,39] have demonstrated that elevated troponin I is associated with lower ejection fraction, higher systolic pulmonary artery pressure and increased length of hospital stay. Moreover, abnormal troponin T is a predictor of increased risk of HF readmission and mortality [40,41]. These associations have persisted up to 3 years from the index hospitalization. Elevated troponin values at discharge predict an increased risk of HF exacerbation, cardiac death and all-cause mortality. Another interesting study, PROTECT [42], showed that increased troponin levels in serial measurements were good predictors of rehospitalization or death at 60 days.

From all the previously reviewed clinical evidence, see Table 1, the value of troponin-based diagnosis and prognosis emerges in patients with ACS or even in HF patients. However, the accuracy of troponin assays can be compromised when threshold values determined from general population are applied to high-risk patient cohorts. Establishing precise cut-off values for each patient subset is expensive, requires large cohorts and standardization of assays from different manufacturers. Therefore, the need for personal data is key to reduce the event-to-diagnosis-time and therefore mitigate unnecessary myocardium damage. Home-based tools can play a decisive role in this context.

### 3.2. Cost-Effectiveness Analysis of Troponin-Based Strategies

Despite the relevance of the LOD of the assay or the role of having appropriate cut-off values, it is the cost factor that is the ultimate parameter to select the best assay for clinical implementation. In this context, a pay per assay scheme is common in the clinical laboratories.

Regardless of the importance of costs especially in limited budgets like the national healthcare systems, the number of available cost-effectiveness analyses focusing on troponin-based patient management strategies are scarce and heterogenous in the scientific literature, making it difficult to establish general conclusions. Nevertheless, the reviews performed by Westwood M. et al. [43], Kip MMA. et al. [44] and St. John A. et al. [45] are a good starting point. These were complemented by other sources retrieved by the authors, see Table 2. In all of them either the incremental cost-effectiveness ratio (ICER) or the willingness to pay (WTP) indicators were used. The former is an index which allows the comparison of the cost-benefits of two possible interventions and is calculated as the cost difference between them divided by the difference in their effect measured in quality adjusted life years (QALY). The latter refers to the maximum amount an individual is willing to hand over to procure a product, service or technology. Further details of health economics are available as Supplementary Material (see Supplementary Material 2). Figure S2 included in the Supplementary Material section (Supplementary Material 2) summarizes the advantages and disadvantages of the different methods available to evaluate the willingness-to-pay indicator. 

#### 3.2.1. Sensitive vs. High-Sensitive Assays

The value of using s-Tn and hs-Tn troponin-based strategies for the patients attending ED with suspicion of AMI but with a normal (or nondiagnostic) ECG and with no major comorbidities requiring admission, have been compared [35,46]. In particular, the strategies were: (1) s-Tn assay measured at presentation using the 10% CV as the threshold for positivity; (2) s-Tn assay measured at presentation using the 99th percentile threshold; (3) hs-Tn assay measured at presentation using the 99th percentile; and (4) s-Tn assay measured at presentation and 10 hours after symptom onset using the 99th percentile. In this latter case, three additional scenarios were: doctor on demand, twice-daily ward round scenario and once-daily ward round scenario.

The findings concluded that a 10-h troponin level measurement is unlikely to be cost-effective in most scenarios compared to a hs-Tn measurement at presentation. The latter was found to have an ICER below the £20,000/QALY threshold. If the hs-Tn assay is also performed at 3 h in addition to the measurement at presentation, it is even more cost-effective due to its impact on early discharge of the patients. Collison et al. [47] found that the 10-h-Tn testing could be cost-effective compared with rapid rule-out strategies if the threshold was increased to £30,000/QALY. Such increase is associated with having a doctor-on-demand scenario. In other words, having medical staff available 24/7 to early discharge the patients as soon as a negative troponin result becomes available.

Similar findings were concluded in patients with no ACS [46,47,48]. The hs-Tn assays were more cost-effective than s-Tn assays and it increased when the accelerated diagnostic algorithms were included. In contrast, the Canadian Agency for Drugs and Technologies in Health reported that s-Tn was the most cost-effective unless WTP increased to over $119,377 in which case hs-TnT was better. In no scenario, hs-TnI was found to be more cost effective than the others. This contradictory finding may be due to restrictions regarding the approval of hs-Tn assays in Canada at the time of the study. Complementarily, Twerenbold et al. [22] found that implementing hs-Tn assays in an experienced center would not increase the need for additional testing, like the performance of coronary angiography.

#### 3.2.2. Point of Care vs. Standard Care

Several multicenter studies [49,50,51] were performed, focusing on the cost-effectiveness of standard care versus PoC panel assay testing in patients attending ED with AMI suspicion but with normal or nondiagnostic ECG and with no major comorbidities. The PoC solution was based on three biomarkers (myoglobin, CK-mass and troponin I) while standard care included different troponin-based strategies performed at hospital admission at least 12 h after the worst symptoms. The PoC was found unlikely to be cost-effective in the UK national health system based on the differences in the mean cost and mean number of QALYs accrued by patients for a time horizon of 3 months and lifetime. A possible explanation for increasing the costs per test is the loss of the economy scale in this type of solutions [45]. Moreover, this study corroborates the findings of several others [48,49,52] which concluded that a multi-biomarker approach had no additional economic benefits.

However, in a primary care context, the probability of PoC to be cost-effective seems to be high. Kip et al. [44] found under conservative assumptions that the probability of being cost-effective merely reduces from 100% to 97% within the WTP range of €0–€50,000 for every 20,000 patients. Interestingly, if WTP increases beyond this range the probability of being cost-effective substantially decreases (e.g., for a WTP range of €0–€100,000 the probability drops to 70%). Consequently, PoC in primary care is expected to be cost-effective even when considering more stringent scenarios and the national health care systems should aim not to surpass the €50,000 limit to maximize the cost-effectiveness of these solutions.

Based on the evidence from the health economic perspective the results suggest that hs-Tn-based strategies are cost-effective due to their capacity to quickly rule-out or discharge patients attending the ED. More cost-effective if accelerated algorithm (0/1 h or 0/3 h) is included. PoC solutions are only cost-effective in a primary care context because these increase the speed of hospital referrals, reduce the number of false positives attending the hospital and prevent overcrowding the ED. Determining the exact value of either indicators (e.g., ICER and WTP) is complex due to the differences between studies (e.g., different countries, patient cohorts and regulatory aspects) or biased outcomes resulting, in some cases, from insufficient real-life data. Nonetheless, a reasonable starting point could be not to surpass the €50,000 limit per every 20,000 patients or the well-established £20,000/QALY threshold.

## 4. Clinical Evidence of Home-Based or Telemonitoring Solutions in High-Risk Patients

**Figure 4:**
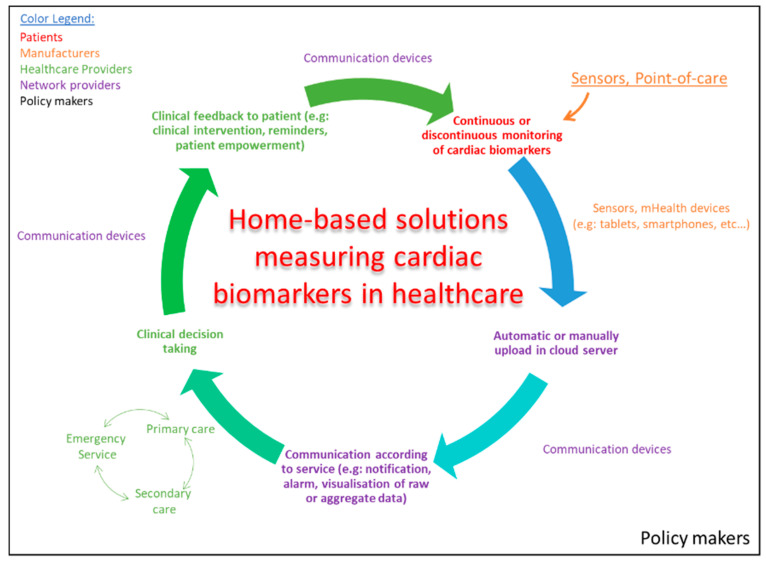
Schematic illustration of the different steps in home-based solutions implemented in current healthcare practices to remotely monitor patients based on cardiac biomarkers and where the different stakeholders contribute the most.

The adoption of home-based solutions by all relevant healthcare stakeholders (e.g., patients, clinicians, healthcare providers, payers and policy makers) will strongly depend on their capability of reducing healthcare expenditures either by reducing the inappropriate use of resources and/or improving patient outcomes. In Figure 4 is schematically represented the role of the different stakeholders in the proliferation of these solutions.

In primary prevention, these tools can positively impact patient outcomes by contributing to patient empowerment [53] and enabling early intervention [54,55,56,57]. A patient aware of his (her) health status can make quicker and wiser decisions, for example preventing unnecessary delays. For every 30-min-delay in receiving intervention, the probability for 1-year mortality of AMI survivors increases by 7.4% [58,59]. In this context, T2DM patients can definitely benefit from remote monitoring tools based on biomarker-guided therapies. They will not only detect in advance cardiac events even if they are asymptomatic, mitigating and reducing myocardial damage, but also help clinicians to stratify their risk of incident CVD up to 10 years in advance [23]. According to the models developed by Seamus et al. [23], after prospectively analyzing 8153 participants without known diabetes or CVD, the chances of suffering incident CVD increases in 2.2 for undiagnosed diabetes patients and in 2.7 for diabetic patients as the troponin levels rise from 6–8 ng/L to ≥14 ng/L. Moreover, independently of omitting the first two or the first five years of follow-up, high troponin values (≥14 ng/L) have also shown their capacity in predicting incidence diabetes (i.e., 1.8 higher chances of incidence diabetes in those patients with normal values of fasting glucose and in 1.45 in those without metabolic syndrome). Early identification of patients at higher risk for incident diabetes and CVD is expected to improve patient outcomes and reduce health care expenditures.

**Figure 5:**
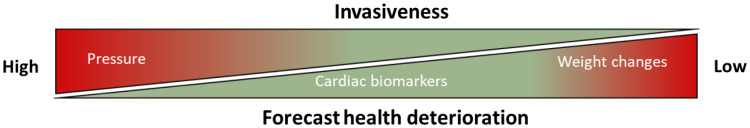
Schematic representation of the invasiveness and the potential for early intervention of different remote monitoring strategies.

Regarding secondary prevention or disease treatment, risk monitoring with home-based solutions was also shown to be very effective in improving quality of life (QoL), in reducing recurrent events, rehospitalization and all-cause mortality of cardiac patients [60,61,62,63,64]. Out of all CVD patients, those suffering from HF are the ones with the highest associated healthcare expenditure. Current interventions range from highly invasive, consisting of implantable pressure sensors [65], to noninvasive solutions based on weight changes, monitoring of ECG signals or a combination of both. Unfortunately, the capability for early clinical intervention is inversely related with the degree of invasiveness (see Figure 5).

Monitoring the levels of key cardiac biomarkers from blood samples has a great potential to become a popular home-based solutions due to: (i) low degree of invasiveness, does not require implantable sensor or surgical procedure, (ii) easy implementation in current clinical practice both in the primary and secondary care setting and (iii) potential anticipation of health deterioration triggering early intervention. A recently published paper [62] has presented the results of a 6-month telemonitoring program using biomarker guided-therapy. In this study of 315 HF patients, improved outcome metrics included a 50% decrease in HF-hospitalizations and a 24% reduction in all-cause hospitalization, a 59% reduction in cardiac biomarker levels and a meaningful improvement of the patients’ QoL, assessed by questionnaires. These findings were corroborated by the Telemedicine Interventional Management in Heart Failure II (TIM-HF2) trial [64] in which 1538 HF patients were retrospectively studied over a 12-month follow up period. Interestingly, this study also tried to evaluate the amount of time saved after the implementation of these programs. To do so, the authors calculated the effort time as the sum of time required per patient to perform medical and nonmedical interventions. The findings highlight that this type of biomarker-guided remote monitoring intervention, could save up to 150 h effort/year for every 100 patients. Even though it does not seem a big saving, the results suggest that these tools can be a good ally of the health care system to manage HF patients, expected to increase due to the continuous up-rise of CVD.

According to the available evidence, home-based solutions can contribute to more efficient care at all levels: for primary care, through fast patient referral when AMI or ACS is suspected, for hospitals by allowing swift and safe rule-out or discharge with later follow-up in a secondary care setting [66]. However, more clinical trials and efforts are needed to continue building an extensive and robust body of knowledge.

## 5. Fostering mHealth and eHealth Solutions

Efforts in designing a convenient regulatory framework and continuous progress in enabling technologies (e.g., infrastructure, artificial intelligence and sensing technologies) are facilitating the development of m-Health and e-Health based solutions.

Regarding the regulatory environment, a recent survey performed by the World Health Organization [67], reveals that out of all the member states, only 58% have an eHealth policy, 55% have legislation to protect electronic patient data and 87% have at least one or more national initiatives to promote eHealth and mHealth services. In Europe, massive progress has been achieved since the early 1990s when the European Commission started to promote the adoption of eHealth practices in the member states. The first eHealth Action plan covered the period between 2004–2012 and has been replaced by the second eHealth Action plan 2012–2020 [68] and complemented by the Digital Agenda for Europe, the Innovation Union initiative and the Digital Single Market Strategy. The key elements are the Directive on Data Protection (Directive 2016/679/EU) [69] which has evolved from the Directive 95/46/EC and the Directive on the application of patients’ rights in cross-border healthcare (Directive 2011/24/EU) [70] and the Medical Devices Regulation (EU) 2017/745 [71]. Despite all these efforts there is still a demand for more policies that can foster the development and implementation of home-based solutions in the healthcare ecosystem.

As to the enabling technologies, it is worth differentiating between the advances in infrastructure, artificial intelligence (AI) and sensing. On one hand, the advances in the network capability through the 5G technology will increase the number of devices connected to the network and the storage capability. The speed of data transfer will increase by reducing the latency time. On the other hand, progress in AI and machine learning (ML) algorithms, embedded in proper sensors or as software as a service (SaaS), are expected to transform a simple wearable solution into a smart tool capable of performing numerous tasks [72,73] without user intervention. As a result, the Food and Drug Administration (FDA) and the European Commission through the Medical Directive Regulation (MDR) are considering basing the regulatory framework on the total product lifecycle. Currently the FDA has approved numerous AI algorithms, focusing on the processing of images and biosignals, covering a wide spectrum of clinical entities [74].

The progress in sensing technologies for wearable and implantable sensors for medical applications is moving forward quickly. Multiple home-based solutions targeting CVD are currently under development, validation, or clinical use. Most of them have employed unobtrusive sensors (e.g., camera, electrodes, photoplethysmography, accelerometer, capacitive sensors, oximeter, microphone, global position system, pressure, gyroscope, thermometer) in order to monitor physiological and behavioral signals [72,73,75,76,77]. These can be active (i.e., emitting energy to the human body and then detecting the reflected or backscattered energy from it) or passive (i.e., detecting the energy emitted by the human body). Sensors can be incorporated in clothing or accessories, in furniture, appliances and construction. However, a limited number of solutions have been developed to monitor biomarkers found in biological fluids (e.g., blood, sweat, tears, saliva or interstitial fluid) [78].

The vast majority of these biomarkers are not measurable without direct chemical detection, in other words, a chemical sensor must come in contact with the sampled fluid containing the analyte. Sampling in a non-invasive or minimally invasive manner still remains a challenge and obtrusive sensors are required. Chemical-to-optical and chemical-to-electrical signal transduction are the two predominant technologies for this purpose. While both of them are cheap, simple and have high miniaturization capabilities, technologies using chemical-to-electrical signal transduction seem like the most promising ones for future clinical application. Several review papers have analyzed how electrochemical sensing is used for biomarker detection as well as the progress in the field and future trends [79,80,81,82,83,84]. Electrochemical biosensors can be classified as potentiometric or amperometric and can be composed of two or three electrodes. These sensors are capable of determining the levels of analyte present in a sample by respectively measuring the responsive changes in potential or current occurring on their surface. In addition, high specificity for a particular analyte or analytes can be achieved by using procedures like ion-selective detection, enzymatic modalities, or immunoassays. As pointed out by Heinkenfeld et al. [84], the detection limits of amperometric sensors seem unsuitable for the measurement of troponin concentrations around nanograms/L. Nevertheless, the continuous development of new conductive materials (e.g., graphene) and nanomaterials (e.g., carbon nanotubes) might minimize or mitigate this limitation.

## 6. mHealth and eHealth Solutions for Detection of Biomarkers in Future Healthcare Systems

The reviewed evidence has shown that home-based biomarker-guided therapies have clinical and economical benefit in the current traditional healthcare systems. However, these are expected to increase as future healthcare systems shift towards a more personalized and patient-centered paradigm. In this context, the fields of patient management and diagnosis offer enormous future opportunities to develop new mHealth and eHealth solutions and services.

According to Frangogiannins et al. [85] there is a need to develop new tools for early and accurate diagnosis of CVD. In this context the development of early alarm systems to detect myocardial injury through the detection of key cardiac biomarkers seems a promising future trend. They can improve patient outcomes by mitigating loss of cardiomyocytes and can be implemented as a diagnostic or as a patient management tool. Besides improving patient outcomes, quick ruling-out of patients from ED will result in additional savings. The use of expensive and bulky laboratory equipment can be replaced by personalized point-of-care testing, avoiding diagnostic delays and ED overcrowding. Moreover, integrating enabling technologies like AI and connectivity features (i.e., bluetooth, wifi and/or gsm) would enhance the safety of the diagnosis and clinical decision making as well as offering personalized online consultation with a dedicated nurse or physician. When applied to high-risk patient cohorts these tools can drastically reduce the event-to-diagnosis delay. Prolonged delays are associated with greater extent of myocardial damage, hence increased risk of early fatality or later progression to HF. Only in the US, one sixth of the people suffering AMI die before hospitalization [58,59]. Individualized pharmacological treatment by developing personalized drug choices and dosing strategies can prevent acute myocardial damage during chronic stages of the disease [86].

Shifting from the traditional to a personalized healthcare system characterized by a proliferation of home-based care solutions, demands the availability and implementation of robust tools. Even though electrochemical immuno-sensing is a promising technology having recently transitioned from bench to bedside [79], further improvements are necessary in effective sampling and transport of biofluids to ensure good reproducibility and avoid contamination.

## 7. Conclusions

Clinical and economical evidence suggests that home-based solutions have a high potential to mitigate or reduce the expected rise of costs in diagnosing and treating ACS as a consequence of the continuous rise of CVD, other chronic disease and life expectancy. When applied to primary and secondary prevention, home-based biomarker strategies for diagnosis and treatment can improve patient outcomes and quality of life by reducing recurrent events, rehospitalization, progression to HF and all-cause mortality of patients with CAD. As a result, home-based solutions can also save costs by contributing to rapid and safe rule-out of patients in secondary care or to expeditious hospital referral in primary care. The ICER or the WTP should not be higher than €50,000 limit per every 20,000 patients or the well-established £20,000/QALY threshold.

In addition, cost-effectiveness is enhanced when applied to high-risk patients who are exposed to higher event rates.

## Figures and Tables

**Table 1:** Summary of the review process regarding diagnosis and prognosis of troponin in high-risk patients.

Patient Cohort.	Studies	Key Characteristics	Major Outcomes
Chronic Kidney Disease (CKD)	Michos E.D. et al. [19]	Examined 124 studies to evaluate diagnosis and prognosis of troponins.	Elevated cTnI or cTnT are potent predictors of mortality in CKD patients with and without suspicion of ACS and independent of the condition whether the patient is receiving dialysis or not.
	Stacy S.R. et al. [20]	Evaluated 23 trials where troponin levels were measured in CKD cohorts.	Identify optimal cut-off points of troponins for patients with CKD and ACS for proper risk stratification.
	Skadberg O. et al. [21]	Serum samples were collected from 20 patients before and after 10 consecutive HD treatments using hs-cTnT.	Need to determine specific cut-off points, before and after hemodialysis, when using hs-Tn assays.
	Twerenbold R. et al. [22]	Multicenter study with 2813 patients with 16% prevalence of renal dysfunction.	Hs-Tn assays showed slightly lower diagnosis accuracy at presentation in CKD patients versus patients without renal dysfunction, no systematic superiority was found between hs-Tn and s-Tn assays and hs-Tn loses specificity when comparing CKD with normal renal function patients.
Diabetes (T2DM)	Whelton S.P. et al. [23]	ARIC trial where 1500 patients with T2DM were recruited.	Troponin testing using hs-Tn assays could be a good biomarker for personalized medicine. Abnormal troponin levels can predict the CVD risk of T2DM patients 10 years in advance.
	Ferdinand K.C. et al. [24]	EXAMINE trail where 3808 patients with T2DM were recruited.	Elevated troponins can help to identify patients at extreme risk for cardiac events. This is a new category identified in 2017 by the Association of Clinical Endocrinologists and American College of Endocrinology on the Comprehensive T2D Management Algorithm.
	Thygesen K. et al. [25]	Summary of meta-analyses.	Hs-Tn have comparable diagnostic and prognostic performance as s-Tn in most clinical settings.
Elderly	Sedighi S.M. et al. [27]	6977 medical records aged ≥65 years without acute coronary events were recruited.	Troponin values in geriatric population is a consequence of non-cardiovascular comorbidities. These results confirm the conclusions of Park KC et al. [26].
	Reiter M. et al. [28]	1098 consecutive patients with symptoms suggestive of AMI where 37% had more than 70 years old.	Elderly NSTEMI patients, using the uniform assay-specific 99th percentile for STEMI diagnosis, were shown to have limited diagnostic capability due to reduced specificity.
	Ichise T. et al. [29]	355 consecutive patients with mean age 66 ± 16.1 years patients attending Kanazawa University Hospital.	When measuring hs-cTnT careful assessment are needed in elderly subjects.
	Zhang S. et al. [30]	679 geriatric inpatients without ACS.	Hs-cTnT elevation caused by non-ischemic acute conditions was very common in geriatric hospitalized patients. Further studies are needed to establish age-specific 99th percentile values of hs-cTnT for elderly individuals.
	Gore M.O. et al. [31]	Data included from three well characterized population-based studies: the Dallas Heart Study (DHS), the Atherosclerosis Risk in Communities (ARIC) Study and the Cardiovascular Health Study (CHS).	Use of a uniform 14 ng/L cutoff for the hs-cTnT assay may lead to overdiagnosis of myocardial infarction, particularly in men and the elderly. Clinical validation is needed of new age- and sex-specific cutoff values for this assay.
	Rains M.G. et al. [32]	Review paper of how different biomarkers were used to diagnose ACS in elderly population evolve.	Desirable to have algorithm specific.
Heart Failure (HF)	Fonarow G.C. et al. [36]	ADHERE study where 65,180 patients were recruited.	Higher troponin levels were associated with higher in-hospital mortality.
	You J.J et al. [37]	EFFECT study where 2000 patients hospitalized in Ontario (CANADA).	Troponin value greater than 0.5 μg/L during the first 48 h of hospitalization was a predictor of increased 1-year all-cause mortality.
	Parenti N. et al. [38]	99 patients discharged from the department between March and December 2002 with a HF diagnosis and samples of cTnI. Patients with acute coronary syndromes, myocarditis or renal failure were excluded.	Elevated troponin I is associated with lower ejection fraction, higher systolic pulmonary artery pressure and increased length of hospital stay.
	Vechia L.L et al. [39]	Thirty-four patients were examined. Upon admission, we measured serum levels of cTnI by conventional immunoenzymatic assay.	cTnI is detected in the blood of 25% to 33% of patients with severe heart failure; its presence may help to identify a high-risk sub-group who faces very poor short-term prognosis.
	Del Carlo C.H. et al. [40]	70 patients with chronic HF worsening that needed hospitalization were studied.	Abnormal troponin T is a predictor of increased risk of HF readmission and mortality.
	Perna E.R. et al. [41]	One hundred and eighty-four consecutive patients with ADHF were enrolled in the absence of an acute coronary syndrome.	Troponin T was an independent long-term prognostic marker of morbidity and mortality and it suggests a role of biochemical risk stratification in HF.
	O’Connor C.M et al. [42]	PROTECT study.	Increased troponin levels in serial measurements were good predictors of rehospitalization or death at 60 days.

cTnI: cardiac troponin isoform I, cTnT: cardiac troponin isoform T, ACS: acute coronary syndrome, hs-cTnT: high sensitive cardiac troponin isoform T, hs-Tn: high sensitive troponin assays, S-Tn: sensitive troponin assay, CVD: cardiovascular disease, NSTEMI: non-ST-segment elevation myocardial infarction, STEMI: ST-segment elevation myocardial infarction.

**Table 2:** Summary of the review process regarding cost-effectiveness of troponin-based strategies.

References	Key Characteristics	Major Outcomes
Westwood M. et al. [43]	Review paper including 18 studies including research registers and conference proceedings up to October 2013.	High-sensitivity assays may provide an effective and cost-effective approach to early rule-out AMI patients. Further research is needed to clarify optimal diagnostic thresholds and testing strategies.
Kip MMA et al. [44]	Patient-level simulation model representing a hypothetical cohort of Dutch population (>35 years) consulting GP with chest complaints.	Point of care troponin strategy is likely to be cost-effective because it reduces hospital referrals in non-ACS patients. Slight increase in nonreferral among ACS patients but with negligible overall health effects.
St. John A. et al. [45]	Review paper on economic assessment of point-of-care testing.	Cost-effectiveness studies in point-of-care testing are limited. There is a need for better understanding of care pathways and how they will change with the introduction of point-of-care testing.
Thokala P. et al. [46]	Patients attending acute hospitals in UK with suspect NSTEMI or unstable angina with no major comorbidities requiring admission.	Delayed troponin testing is unlikely to be cost-effective compared with high-sensitivity troponin testing at presentation in most scenarios.
Collison P.O et al. [47]	Sub-study of point-of-care arm of the RATPAC trial taking place in the emergency department of six hospitals.	The measurement of high-sensitivity cardiac troponin is the best single marker in patients presenting with chest pain. Additional measurements of myoglobin or CK-MB are not clinically effective or cost-effective. The optimal timing for measurement of cardiac troponin remains to be defined.
Jülicher P. et al. [48]	Part of ADAPT trial in Australia-New Zealand of 938 patients who presented at an adult Emergency Department of a tertiary referral hospital with at least 5 min of symptoms suggestive of acute coronary syndrome.	High-sensitivity troponin I algorithms are likely to be cost-effective on a hospital level compared with sensitive troponin protocols.
Goodacre S. et al. [49]	Systematic review, meta-analysis and economic modelling of diagnostic strategies suspected of acute coronary syndrome.	Although presentation troponin has suboptimal sensitivity, measurement of a 10-h troponin level is unlikely to be cost-effective in most scenarios compared with a high-sensitivity presentation troponin.
Goodacre S. et al. [50]	RATPAC trial. Multicenter pragmatic open randomized controlled trial and economic evaluation of point-of-care testing used in six acute hospital emergency departments in UK.	Point-of-care testing is more expensive than standard care and unlikely to be considered cost-effective.
Fitzgerald P. et al. [51]	The RATPAC trial a multicenter individual patient randomized controlled trial comparing diagnostic assessment using a POC biomarker panel (CK-MB, myoglobin and troponin, measured at baseline and 90 min) to standard care without the POC panel in 2243 patients.	Point-of-care panel assessment does not reduce costs despite reducing admissions and may even increase costs. It is unlikely to be considered a cost-effective use of health care resources.

## Supplementary Materials

The following are available online at https://www.mdpi.com/1424-8220/20/17/5006/s1, Figure S1: Number of references used in the manuscript according to the type of document, Table S1: Items reviewed to assess the quality of the clinical guidelines and consensus documents, Figure S2: Comparative evaluation of competing methods for measuring willingness-to-pay.
