# Supplementary material for: Benefits of Home-Based Solutions for Diagnosis and Treatment of Acute Coronary Syndromes on Health Care Costs: A Systematic Review
Source: Sensors (Basel). 2020 Sep 3;20(17):5006. doi: 10.3390/s20175006 (PMC7506920; doi:10.3390/s20175006)
Supplement: Supplementary file 1 [file sensors-20-05006-s001.pdf]

sensors

Benefits of Home-Based Solutions for Diagnosis and Treatment of Acute Coronary Syndromes on Health Care Costs: A Systematic Review

Pau Redón [1,2,*], Atif Shahzad [2], Talha Iqbal [2] and William Wijns [1,2,3]

[1] CÚRAM Center for Research in Medical Devices, H91 W2TY Galway, Ireland; william.wijns@nuigalway.ie
[2] Smart Sensor Lab, School of Medicine, National University of Ireland, Galway (NUIG), H91 TK33 Galway, Ireland; atif.shahzad@nuigalway.ie (A.S.); t.iqbal1@nuigalway.ie (T.I.)
[3] Saolta University Healthcare Group, University Hospital Galway, Newcastle Road, H91 YR71 Galway, Ireland
* Correspondence: pau.redonlurbe@nuigalway.ie

Supplementary Material Regarding Methodology (S1):

The manuscript entitled "Benefits of Home-Based Solutions for Diagnosis and Treatment of Acute Coronary Syndromes on Health Care Costs: A Systematic Review" is the result of combining a wide variety of available published research studies, reviews and legislation focusing on sensing technologies currently available for home-based telemonitoring systems; relevance of personal data for diagnosis and prognosis of acute coronary syndromes (ACS) including NSTEMI (non-ST-elevation myocardial infarction), unstable angina and STEMI (ST-segment elevation myocardial infarction); anticipated clinical benefits from implementation of telemonitoring in this context; and features that make an ambulatory troponin-based strategy potentially cost-effective.

A wide variety of documents were finally included. These were classified as: (i) Consensus document / Clinical guidelines, (ii) Review papers, (iii) Original Research papers, (iv) Fact sheets / Web sites, (v) Legislation. Figure S1 summarizes the number of references pertaining to each of the groups. Interestingly, this manuscript incorporates de findings of more than 15 consensus documents or clinical guidelines and only 25 out of a total of 86 references were review papers.

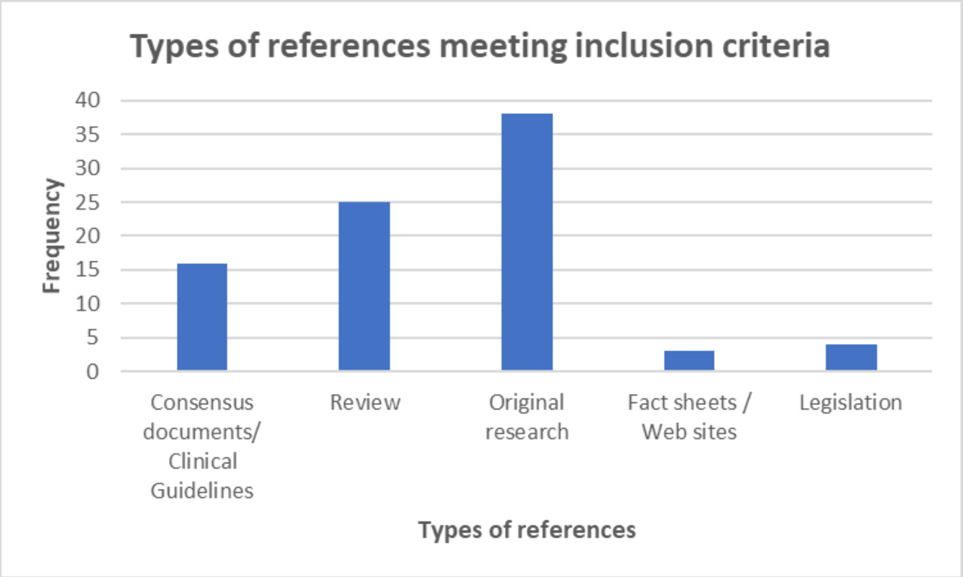

Figure S1. Number of references used in the manuscript according to the type of document.

The PRISMA methodology was used to perform this systematic review in a methodological manner and complemented, if necessary, with the AGREE-II methodology (Brouwers et al., 2010) to evaluate the quality of clinical guidelines and consensus documents as well as clinical trials. The tool

is specifically oriented to these documents and comprises 23 items organized into 6 quality domains: scope and purpose, stakeholder involvement, rigour of development, clarity of presentation, applicability and editorial independence. Each item must be rated from 1 (strongly disagree) to 7 (strongly agree) and the final score is calculated for each domain and represented as a percentage of the maximum possible score. In Table S1 are listed the items used to evaluate the different documents.

Table S1. Items reviewed to assess the quality of the clinical guidelines and consensus documents.

**AGREE-II items**
1.- The overall objectives of the guidelines is (are) specifically described.
2.- The health question(s) covered by the guideline is (are) specifically described.
3.- The population (patients, public, etc..) to whom the guideline is meant to apply is specifically described.
4.- The guideline development group includes individuals from all relevant professional groups.
5.- The views and preferences of the target population (patients, public, etc…) have been sought.
6.- The target users of the guideline are clearly defined.
7.- Systematic methods were used to search for evidence.
8.The criteria for selecting the evidence are clearly described.
9.The strengths and limitations of the body of evidence are clearly described.
10.The methods for formulating the recommendations are clearly described.
11.The health benefits, side effects, and risks have been considered in formulating the recommendations.
12.There is an explicit link between the recommendations and the supporting evidence.
13.The guideline has been externally reviewed by experts prior to its publication.
14. Procedure for updating the guideline is provided
15. The recommendations are specific and unambiguous.
16. The different options for management of the condition or health issue are clearly presented
17. Key recommendations are easily identifiable.
18. The guideline describes facilitators and barriers to its application.
19. The guideline provides advice and/or tools on how the recommendations can be put into practice.
20. The potential resource implications of applying the recommendations have been considered.
21. The guideline presents monitoring and/or auditing criteria.
22. The views of the funding body have not influenced the content of the guideline.
23. Competing interests of guideline development group members have been recorded and addressed.

The application of this tool to the clinical guidelines and consensus documents has revealed interesting findings. In many of them, the authors did not describe the process followed to gather the evidences. The impact of this limitation is mitigated by the fact that the documents were developed by prestigious scientific associations, from Europe and USA, where key opinion leaders wrote and reviewed the document prior to publication. In addition, the conclusions and recommendations of these documents are considered as good clinical practices and all authors' interests were listed in the Section Conflict of Interest.

On the other hand, the major limitations observed in some clinical studies included in this section were mainly related with their heterogeneity. A closer analysis revealed the following limitations: i) testing a limited number of troponin assays and extrapolating the results to those of other manufacturers, ii) small sample size or unevenly distributed cohorts according to age, gender, race and ethnicity, iii) lack of time data between event and troponin reading or short follow-up periods and iv) not identified confounding associations.

Limitations were also manifested when reviewing the limited number of cost-effectiveness (CE) studies analysing troponin-based strategies and those presenting clinical evidences of home-based tools tested in cardiac patients in a real clinical context. On the CE studies the limitations focused on the heterogeneity between them due to differences between countries (e.g: legislation, product availability, implemented procedures, costs, etc...), selection bias (e.g: inappropriate exclusion of patients and patient follow up) and outcome bias (e.g: responder bias due to significant differences in patient participation from one intervention arm in comparison with the other and the lack of real-life data which requires conservative assumptions when performing health economics simulations).

Regarding evidences in telemonitoring strategies the limitations focused on heterogeneity between the studies and the lack of economic data. This could be useful to compare the costs between telemonitoring strategies and standard care.

Despite the number of limitations, these can be reasonably common in a real clinical context and highlights the heterogeneity of scenarios when it comes to the diagnosis and treatment of acute coronary syndromes. In this context is where home-based solutions, capable of offering personalised and properly contextualised data, are more relevant and where personalised medicine becomes more crucial.

Supplementary Material Regarding Health Economic Indicators (S2)

The following section describes some of the basics of health economics taking the paper of Cohen et al. (Cohen & Reynolds, 2008) as baseline and complementing it from different literature sources (Breidert et al., 2006; Prieto & Sacristán, 2003; Shiroiwa et al., 2013; Takura, 2018).

Several types of economic studies coexist in the health economic field. These are: cost-minimization, cost-effectiveness ratio (calculates the incremental cost in units of currency while expressing clinical benefits in nonmonetary terms such as life-years gained or adverse events avoided) and cost-utility analyses (estimates the effectiveness using measures that reflect individual or societal preferences for differing health states, such as quality-adjusted life years), cost-benefit, "what if" analysis and cost-consequences analysis. Equation 1 summarises how the cost-effectiveness ratio is calculated. This is by the far the most popular tool.

Cost-effectiveness = (Cost_new – Cost_ref) / (Effectiveness_new – Effectiveness_ref) (Eq. 1)

Generally speaking, their objective is to allow clinicians and policymakers to make more rational decisions regarding clinical care and resource allocation of new drugs, tests, procedures and devices with respect to standard care. There is also the incremental cost-effectiveness ratio (ICER), a concept that can be utilized in health technology assessments where the increased costs are compared with the incremental increase in effects (incremental increase costs/ incremental increase in effect). The concept is that if one technology has higher costs but higher benefits then the so-called performance will improve (Takura, 2018).

The majority of the analyses listed previously, the associated costs of new therapies, drugs, services or devices must be calculated. In some cases, this can be complex but by far less challenging than evaluating effectiveness. Under certain circumstances this effectiveness might be changes in life expectancy while in others can be the avoidance of adverse events or improvement in quality of life (QoL). QoL and quality adjusted life year (QALY) are the preferred measures. Focusing on the latter, it assumes that a year of life lived in perfect health is worth 1 QALY and that a year of life lived in a state of less than this perfect health is worth less than 1. Consequently, these are calculated by multiplying years of life by utility (health state) (Prieto & Sacristán, 2003). Researchers often use indirect measures like EuroQol or the Medical Outcomes Study Short-Form.

CE studies can be interpreted from an analytic perspective or through incremental comparisons. The former, focuses on understanding the global balance between societal costs and societal benefits, however, some argue that a fully transparent accounting of CE should demonstrate explicitly the effect on each of the individual stakeholders. The latter is based on incremental comparisons where each relevant strategy is compared with the next best alternative, based on the economic concept of opportunity costs.

To extract the most out of these economic analyses it is key to understand their potential limitations. The first of them is time horizon. This should be long enough to take into consideration all the effects on either clinical or economic outcomes. The second is uncertainty. In this case, generally is described as confidence intervals, p-value or power and sensitivity analyses can help to evaluate it. The third is that this studies are not well suited for clinical decision making at an individual patient level nor for making complex resource allocation decisions because it cannot include all of the values (e.g: equity, feasibility or overall budgetary impact). The fourth is the concern about the accuracy and transparency of CE data. Regulators outside US are increasingly requiring budget impact analyses along with CE studies when assessing new therapies. Nevertheless, despite these limitations, CE analyses are expected to continue growing in importance.

On the other hand, there are some scenarios where it is not possible to measure QALY, express costs and benefits in market prices or difficult to evaluate severity (Shiroiwa et al., 2013). In these cases, the willingness-to-accept (WTA) or the willingness-to-pay (WTP) indicators can be used. In the former, is where patients state the minimum amount they would need to be compensated in order to forgo a good or service while the latter is defined as the monetary amount an individual is worth offering to acquire a new treatment, drug, service or device. The EuroVAQ study measured WTP values per QALY in nine European countries and the results suggested that the WTP per QALY for

worse health state is higher than that for other states. Even though more empirical studies are needed to evaluate this relationship, WTP might help to improve decision-making. There are some methods to measure the WTP, like: based on market data analysis, experiments (e.g: laboratory experiments, field experiments, auctions), direct surveys (e.g: expert judgement, customer surveys) and indirect surveys (e.g: conjoint analysis, discrete choice analysis) (Breidert et al., 2006). A comparison of pros/cons between each of them can be observed in Figure S2 (Breidert et al., 2006).

Methods	Cost effective	Time efficient	Flexibility price/product combinations	Validity of estimations	Real purchase behaviour	Observed choice behaviour	Individual level estimations
Market data	≈	≈	✗✗	✓✓	✓✓	✓✓	≈
Experiments	✗✗	✗✗	✓✓	≈	≈	✓	≈
Direct survey	✓✓	✓✓	≈	✗✗	✗✗	✗✗	✓✓
Conjoint analysis	✓	✓	✓✓	✓	✗✗	✗✗	✓✓
Discrete choice analysis	✓	✓	✓✓	✓	✗✗	✓	✓

✓ Advantage ✗ Disadvantage ≈ No clear advantage or disadvantage

Figure S2. Comparative evaluation of competing methods for measuring willingness-to-pay. Adapted from (Breidert et al., 2006).

References of Supplementary Materials (S1 and S2):

1. Breidert, C., Hahsler, M., & Reutterer, T. (2006). A Review of Methods for Measuring Willingness-to-Pay. Innovative Marketing.
2. Brouwers, M., Kho, M., Browman, G., Burgers, J., Cluzeau, F., Feder, G., Fervers, B., Graham, I., Grimshaw, J., Hanna, S., Littlejohns, P., Makarski, J., & Zitzelsberger, L. (2010). AGREE II: Advancing guideline development, reporting and evaluation in healthcare. CMAJ: Canadian Medical Association journal = journal de l'Association medicale canadienne, 182, E89-842.
3. Cohen, D. J., & Reynolds, M. R. (2008). Interpreting the Results of Cost-Effectiveness Studies. Journal of the American College of Cardiology, 52(25), 2119-2126. https://doi.org/10.1016/j.jacc.2008.09.018.
4. Prieto, L., & Sacristán, J. A. (2003). Problems and solutions in calculating quality-adjusted life years (QALYs). Health and Quality of Life Outcomes, 1, 80. https://doi.org/10.1186/1477-7525-1-80.
5. Shiroiwa, T., Igarashi, A., Fukuda, T., & Ikeda, S. (2013). WTP for a QALY and health states: More money for severer health states? Cost Effectiveness and Resource Allocation : C/E, 11, 22. https://doi.org/10.1186/1478-7547-11-22.
6. Takura, T. (2018). An Evaluation of Clinical Economics and Cases of Cost-effectiveness. Internal Medicine, 57(9), 1191-1200. https://doi.org/10.2169/internalmedicine.9835-17.
